# The Solution to Sustainable Eating Is Not a One-Way Street

**DOI:** 10.3389/fpsyg.2020.00531

**Published:** 2020-03-24

**Authors:** Charlotte Vinther Schmidt, Ole G. Mouritsen

**Affiliations:** Department of Food Science, Taste for Life and Design and Consumer Behavior, University of Copenhagen, Frederiksberg, Denmark

**Keywords:** food, sustainable eating, diet, holistic approach, taste, umami, flexitarian, vegetables

By 2050 the Earth has to feed a population approaching 10 billion. The recent report from the EAT-Lancet Commission on Healthy Diets from Sustainable Food Systems (Willett et al., 2019) documents that the conditions for a sustainable, healthy, and nutritious diet for a growing global population can only be established via an acute and major change in the global food systems. This change involves a diet with much more plant-based food than now, including 500 g vegetables and fruit every day and little or no red meat. It is well-known that most people have difficulties eating that much green. The barriers to eating enough vegetables and fruit may be of both psychological, physiological, social, and cultural nature. In addition, plant-based food is lacking in the basic tastes sweet and umami that humans over evolutionary time scales have been primed to crave (Wrangham, 2009). Without confronting these fundamental facts, we may fail in providing for a more green and sustainable future for the planet. In the present Opinion we propose a solution to meet these challenges by the adoption of a holistic flexitarian approach shaped by fundamental insight into taste, and in particular, how to sustain sweetness and umami in a green diet, using only minimal sources from the animal kingdom.

A sustainable diet may be defined as “*a diet comprised of foods brought to the market with production processes that have little environmental impact, is protective and respectful of biodiversity and of ecosystems, and is nutritionally adequate, safe, healthy, culturally acceptable, and economically affordable*” (Pimentel and Pimentel, 2003; Aleksandrowicz et al., 2016; Chai et al., 2019). This is often exemplified as plant-based diets. The literature typically divides plant-based diets into three major groups being flexitarian, vegetarian, and vegan. Common for these diets is that animal-based food, seen in an omnivore diet, is substituted to a smaller (flexitarian), larger (vegetarian), and absolute (vegan) extent. In recent years, an increasing interest in eating more sustainable has been observed and more people are eating a vegetarian and flexitarian diet (Horseman, 2019; Yougov.com, 2019) and sustainability is even described as a megatrend (Mittelstaedt et al., 2014; Hale, 2018). Thus, it may appear that academia (Reijnders and Soret, 2003; Scarborough et al., 2014; Van Kernebeek et al., 2014) as well as some first-mover consumers, are agreeing that meat is not sustainable. However, it must not be forgotten that meat comes in various degrees of sustainability. In addition, meat may often be strongly linked to social and cultural traditions, and it has a high nutritional content and umami taste. Cutting it out of the diet completely may, therefore, pose some significant challenges for some consumers.

The EAT-Lancet report (Willett et al., 2019) points out that the global food systems in the Anthropocene are the main reason for the changes in the Earth's ecosystems, including climate changes. Agriculture is responsible for using 40% of the land (Foley et al., 2005), 30% of greenhouse emissions (Vermeulen et al., 2012), and 70% of freshwater use (Steffen et al., 2015). Fisheries have fully exploited 60% of the wild stock, overfished another 30%, and the global catch has declined in recent decades. The cost of this type of food production has been a great loss in biodiversity, damage to whole ecosystems, as well as the emission of excess nutrients and greenhouse gasses. In addition, crucial global cycles of carbon, phosphorus, and nitrogen have been disturbed. At the same time, food waste from production to consumption is skyrocketing. By adding to this that 820 mio. people are starving and 2 billion people now suffer from diet-related diseases, it is clear that the current global food systems are neither sustainable nor healthy (Harland and Garton, 2015; Searchinger, 2019; Willett et al., 2019).

Based on detailed calculations and projections, the EAT-Lancet report proposes a diet that allegedly is sustainable, nutritious, and healthy and can take us safely toward 2050. This diet consists of mainly vegetables, fruit, whole grain, legumes, nuts and unsaturated fats, only moderate or small amount of fish and poultry, and no or very little red meat, processed meat, added sugars, refined cereals, and starchy vegetables. With this recommendation, it should be possible to meet the United Nations Sustainable Development Goals (United Nations (UN), 2019). However the solution is fragile, and a small growth in the consumption of red meat or dairy products can have catastrophic effects (Willett et al., 2019).

The Eat-Lancet standard for the daily intake of vegetables and fruit is aligned with many countries' national diet recommendations of 500–600 g greens a day. This brings us to the key question: Can we eat that much green? The general observation is that most people's eating habits do not agree with that much green every day. Even if we know that a more green diet is more healthy and more sustainable, we are not going to eat it in the long run if it does not conform with our taste preferences. This brings us to the next question: is a green diet tasty enough? In the present authors' opinion, this is the most critical question. We believe that there are two fundamental reasons why a green diet may not be to our liking. One has to do with the evolution of our species and the other is related to the biology of plants.

Before we attack the challenge of taste and present a possible solution, it should be noted that there can be personal and contradicting issues with, on the one hand knowing or believing that a green diet is more sustainable and healthy, but on the other hand still not succeeding in eating as recommended. This can lead to chronic feelings of guilt and possibly eating disorders (Brytek-Matera et al., 2019). In addition, it is worrisome that some people, in particular children and young people, blindly force themselves to become vegetarian or even vegans without understanding how to compose and supplement a plant-based diet that lacks essential nutrients. Especially for a vegan diet, super-unsaturated fatty acids, vitamin B_12_ and D_3_, taurine, creatine, and heme iron may lack as well as contain insufficient amounts of iodine and selenium (Kristensen et al., 2016; Petti et al., 2017). A vegan diet may therefore pose a challenge to accommodate the varying dietary needs that exist across the entire life-span, specifically for vulnerable groups such as children, adolescents, elderly, and sick people.

Let's start noting that plants, in general, do not “want” to be eaten. The roots, the stem, the foliage, and unripe fruits are not supposed to be eaten, and that is why they are often bitter, sour, and poisonous in some cases (McGee, 2004). This is a chemical defense system developed by organisms that are unable to run away from an enemy. Only the ripe fruits are meant to be eaten by animals in order for the plant to reproduce. Hence, these fruits are sweet and some of them have umami taste, like the tomato (due to free glutamate). In the absence of muscles, plants do not have as much ATP as animals, and ATP is s source of free nucleotides (e.g., inosinate) that synergistically act with free glutamate to elicit enhanced umami-taste (Mouritsen and Khandelia, 2012). Hence, green plants generally lack sweetness and umami—two basic tastes humans are born to crave.

The reason for this craving is rooted in human evolution (Wrangham, 2009). Sweetness is a signal of calories, and umami a signal of accessible proteins, both factors that are important for survival. Our distant ancestors, the Australopithecines, were fruit eaters, and their craving for sweetness signaled by the aroma of the ripe fruits have stayed with us. However, for more than two million years and increasingly after humans started using the fire for cooking about 1.9 mio. years ago, we have also been meat-eaters (Wrangham, 2009). This energy and protein-dense diet has been a prerequisite for the evolution of our big and energy-consuming brain. In the present context, meat elicits umami taste as it is rich in free glutamate and free inosinate, which we over time have come to associate with deliciousness (Mouritsen and Styrbæk, 2014).

Faced with the challenge of eating 500–600 g greens a day, we are thus confronted with fundamentals of plant biology and human evolution. We are looking at a large quantity of foodstuff that is not really tasty enough. It simply lacks sweetness and umami. But there is a cure for this and that is what cooking and the culinary sciences are about, and it is not a matter of only adding sugar and MSG.

There are several ways of providing a green diet with sweetness and umami. One is to prepare the food such as vegetables by adding natural ingredients that add sweetness (e.g., fruits) and umami (seaweeds, algae, fungi) or by using extracts and fermented products based on plant material (e.g., fermented vegetables and yeast products). This approach will be fully vegan but can be quite cumbersome. Vegetarians have more options by eating eggs and fermented dairy that supply umami. Flexitarians have many more possibilities by supplementing their green diet with marine foods like fish, mollusks, and shellfish as well as taste-intensive fermented sauces of fish and shellfish, foodstuff that all are umami-rich (Mouritsen and Styrbæk, 2014). Little utilized marine resources such as algae and seaweeds can also be excellent sources of umami taste in addition to contributing high nutritional value (Mouritsen, 2013; Mouritsen et al., 2019).

Another way involves technological improvements, using state-of-the-art food processing as a solution to transfer inedible foods and by-products into edible food, converting plant-based ingredients into umami-tasting foods, e.g., by various fermentation techniques (Mouritsen, 2018). However, consumer attitude toward processed foods is rather negative (Foodnavigator.com, 2019) and negative publicity about processed foods is a problem when not building on factual insight into those processing techniques that can ensure sustainability along with food quality and taste (Rego et al., 2017). As for meat, various degrees of sustainability and quality exist when it comes to processing.

Meat of animal origin is the easiest way of obtaining umami. Hence, it becomes an important issue to differentiate various types of animal-based food sources according to the degree of sustainability, and not only their animal-specific origin. This would favor use of animal-based by-products processed by a high-quality production processes designed to optimize umami taste, e.g., traditional garum and soy sauce processing (Mouritsen et al., 2017), under-utilized animal resources, e.g., cephalopods (Faxholm et al., 2018; Mouritsen and Styrbæk, 2018), and specific national invasive animal species as the Pacific oyster in Scandinavia. In this perspective, meat may be consciously chosen and applied as a seasoning rather than the main part of a meal.

Strategic approaches made by private organizations, academia, and the government should apply a holistic approach when it comes to recommending sustainable diets in order to be more successful in getting people to eat more plant-based diets. A holistic approach may encounter multiple measures instead of merely excluding meat and processed foods in general. Such a holistic approach may be supported by the findings by Springmann et al. (2018). These authors investigated several options to obtain a global sustainable food system, including plant-based dietary changes, technological improvements, and reduction of food waste and found that no single measure is enough to keep planetary effects in order simultaneously, but that a synergistic combination would be needed (Driscoll, 2019).

We would advocate a lacto-ovo-vegetarian or flexitarian approach, using animal sources as a minor part of a meal or as a means of seasoning, as the most sensible and realistic way of eating sustainably in order to meet the EAT-Lancet Commission's recommendations since rather small amounts of umami-rich foodstuffs from animals can make large volumes of green food delicious for a large population on a daily basis (EAT, 2020). In this way, more people may be prepared to change their diet since they would not have to replace food with nutritional supplements, change fundamental social and ethnic traditions, and most importantly, they may not have to fight against their evolutionary determined cravings for umami. Social and psychological challenges in changing one's diet toward eating more plant based may be less pronounced when applying these recommendations rather that adopting a strict vegan diet.

It should be remarked, that any change in dietary pattern should conform to the general needs for essential nutrients, e.g., amino acids, vitamins, and super-unsaturated fatty acids, as well-account for the unique nutritional status and requirements of the individual, in particular children, elderly, and people with diseases. Finally it should be recognized that there are a number of other social and psychological factors that influence people's food choice and acceptance, such as gender, moral, and attachment to meat (O'Doherty Jensen and Holm, 1999; Ruby and Heine, 2011; Graça et al., 2015; Spencer et al., 2018).

In our view, sustainable eating has to be built on a holistic approach. Asking a major part of the global population for going vegetarian or even vegan is simply not a realistic option neither in the short or in the long run. Sustainable eating on a global scale is not a one-way street.

## Author Contributions

CS and OM conceived, wrote, and provided approval for publication of this work.

### Conflict of Interest

The authors declare that the research was conducted in the absence of any commercial or financial relationships that could be construed as a potential conflict of interest.
